# Supplementary material for: Genome information processing by the INO80 chromatin remodeler positions nucleosomes
Source: Nat Commun. 2021 May 28;12:3231. doi: 10.1038/s41467-021-23016-z (PMC8163841; doi:10.1038/s41467-021-23016-z)
Supplement: Supplementary file 1 — Supplementary Information [file 41467_2021_23016_MOESM1_ESM.pdf]

Supplementary Information

Genome information processing by the INO80 chromatin remodeler positions nucleosomes

Elisa Oberbeckmann[1,2,#], Nils Krietenstein[1,3,#], Vanessa Niebauer[4,5], Yingfei Wang[6], Kevin Schall[4,5], Manuela Moldt[4.5], Tobias Straub[7], Remo Rohs[6], Karl-Peter Hopfner[4,5]*, Philipp Korber[1]*, Sebastian Eustermann[4,5,7]*

[1]Division of Molecular Biology, Biomedical Center, Faculty of Medicine, Ludwig-Maximilians-Universität München, Martinsried near to Munich, Germany

[2] current address: Department of Molecular Biology, Max Planck Institute for Biophysical Chemistry, Göttingen, Germany

[3] current address: Department of Biochemistry and Molecular Pharmacology, University of Massachusetts Medical School, Worcester, Massachusetts, USA

[4]Gene Center, Faculty of Chemistry and Pharmacy, Ludwig-Maximilians-Universität München, Munich, Germany

[5]Department of Biochemistry, Faculty of Chemistry and Pharmacy, Ludwig-Maximilians-Universität München, Munich, Germany

[6]Departments of Quantitative and Computational Biology, Chemistry, Physics & Astronomy, and Computer Science, University of Southern California, Los Angeles, CA 90089, USA

[6]Core Facility Bioinformatics, Biomedical Center, Faculty of Medicine, Ludwig-Maximilians-Universität München, Martinsried near to Munich, Germany

[7]European Molecular Biology Laboratory (EMBL), Structural and Computational Biology Unit, Heidelberg, Germany

[#]These authors contributed equally.

*Corresponding authors:
sebastian.eustermann@embl.de, pkorber@lmu.de, hopfner@genzentrum.lmu.de

*Corresponding authors: sebastian.eustermann@embl.de, pkorber@lmu.de, hopfner@genzentrum.lmu.de

Table of Contents

Supplementary Figures	1
Supplementary Table 1	6
Supplementary References	7

Supplementary Figures

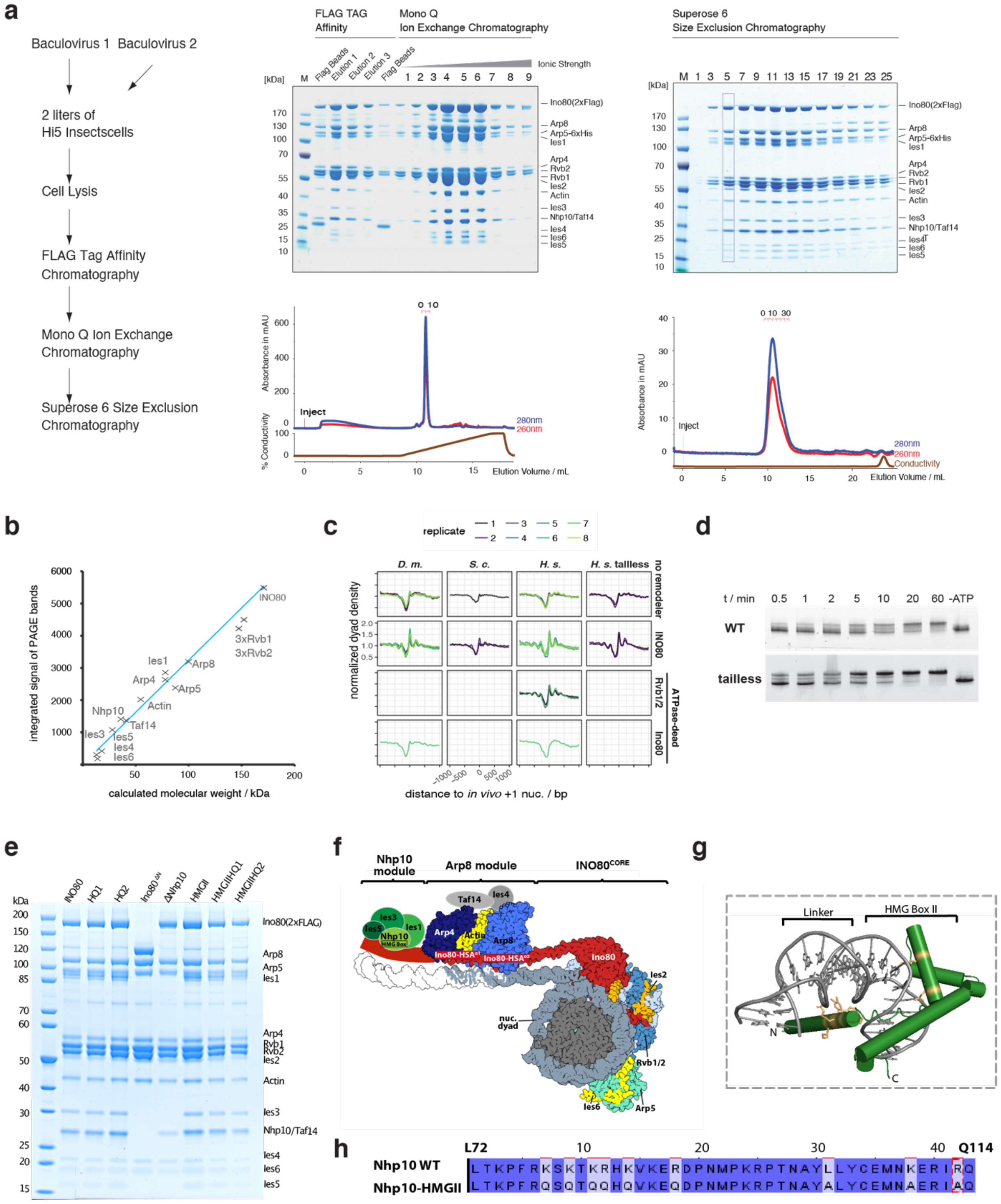

Supplementary Figure 1. Expression, purification and activity of recombinant INO80 a Recombinant expression and purification of 15-subunit *S. cerevisiae* INO80 complex. Left: Schematic of expression and purification work flow. Two baculoviruses encoding five (Ino80, Arp5, Ies6, Rvb1 and Rvb2) and ten INO80 subunits (Ies1-5, Nhp10, Taf14, Actin, Arp4, Arp8), respectively, were used for insect cell expression. Middle and right: representative SDS-PAGE analysis; numbered lanes indicate elution fractions matching chromatograms below gels. Whole genome reconstitutions were performed using biological replicates (n>3) of recombinant wild type and mutant INO80 complexes. **b** Quantification of Coomassie-stained SDS PAGE bands (boxed lane panel a, n=1) showed stoichiometric assembly of recombinant *S. cerevisiae* INO80 complex. Note that AAA^+ ATPase Rvb1 and Rvb2 form a hetero-hexamer, consistent with cryoEM structural studies of human and *Chaetomium thermophilum* INO80 complexes[1,2]. **c** Composite plots as in Figure 2d of MNase-seq data of individual replicates for the indicated

(same abbreviations as in Figure 2b) combinations of histones (columns) and remodeling enzymes (rows). **d** Top: Native gel electrophoresis analysis at indicated time points of mononucleosome sliding assay kinetics with wild type (WT) or tailless (tailless) recombinant *H. sapiens* histones and WT recombinant *S. cerevisiae* INO80 complex. "-ATP" denotes 60 min time point without ATP. **e** SDS-PAGE analysis of purified, recombinant WT (INO80) or indicated mutant complexes. **f** Left: Structure-based[2,3] model of a nucleosome bound by the INO80 complex with indicated submodules and subunits. Taf14, Ies4 and Nhp10 module organization is assumed. **g** Model of Nhp10 HMG box-like and Linker region (residues 62-172) based on TFAM structure (pdb 3tq6). **h** Sequence alignment showing mutated residues in Nhp10-HMGII mutant. Panels e-h are also shown in the accompanying paper[4].

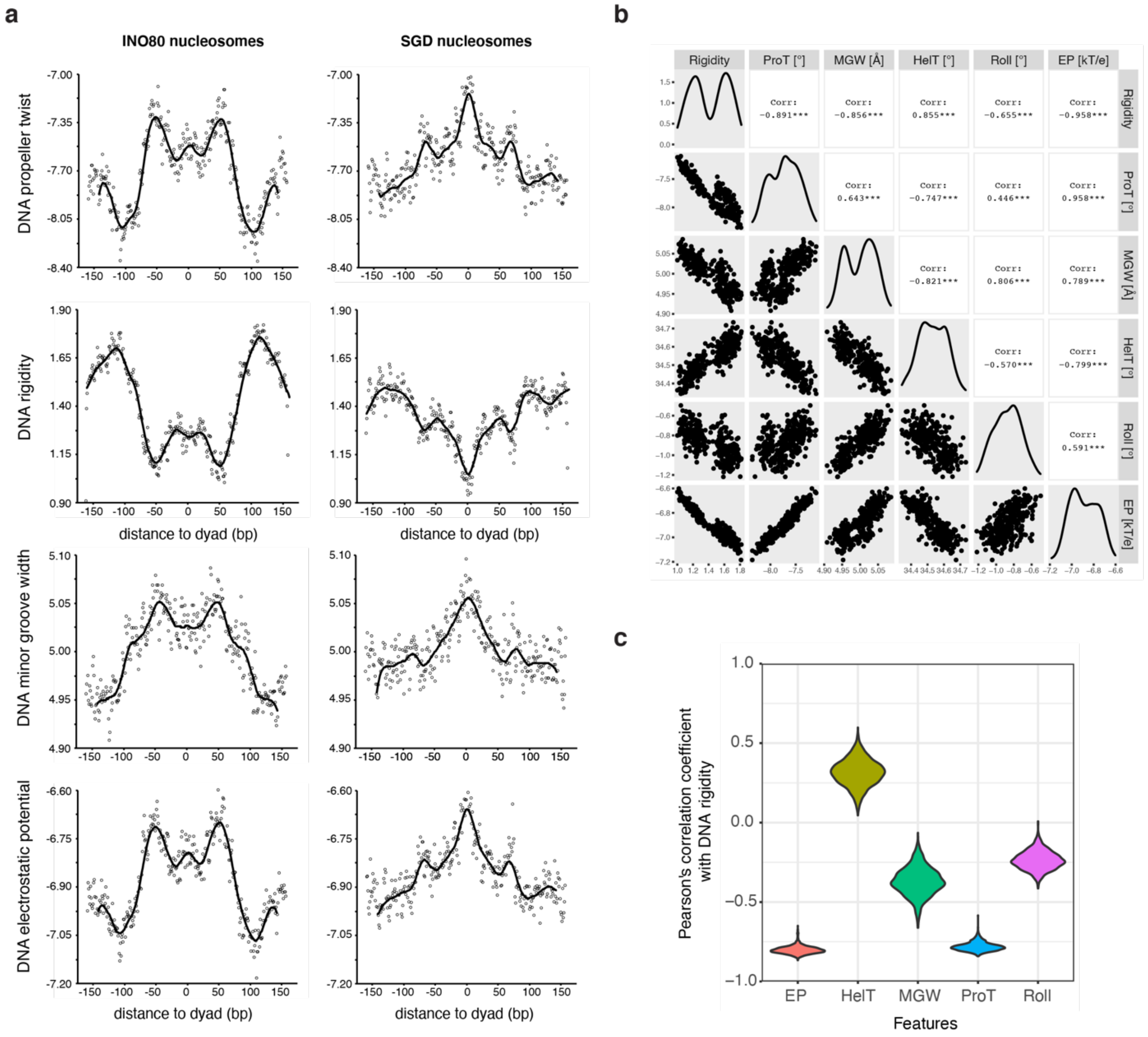

Supplementary Figure 2. DNA shape/mechanics features of INO80- and SGD-positioned nucleosomes. a As Figure 4a, but for the indicated DNA shape (DNA propeller twist, DNA minor groove width, DNA electrostatic potential) and mechanics (DNA rigidity) profiles of INO80- and SGD-positioned nucleosomes. **b** Pearson's correlation coefficients between six DNA feature categories: minor groove width (MGW), helix twist (HelT), propeller twist (ProT), Roll, electrostatic potential (EP), and DNA rigidity. The average profiles of DNA features across all nucleosomal sequences (n=2393 of data as in panel a after filtering of sequences as defined in Figure 3a and methods section) are shown and used to obtain the correlation coefficients between features. **c** Violin plot of Pearson's correlation coefficients between DNA rigidity and other DNA shape features of all nucleosomal sequences (n=2393). The coefficients were obtained by correlating the DNA feature profiles of each sequence individually.

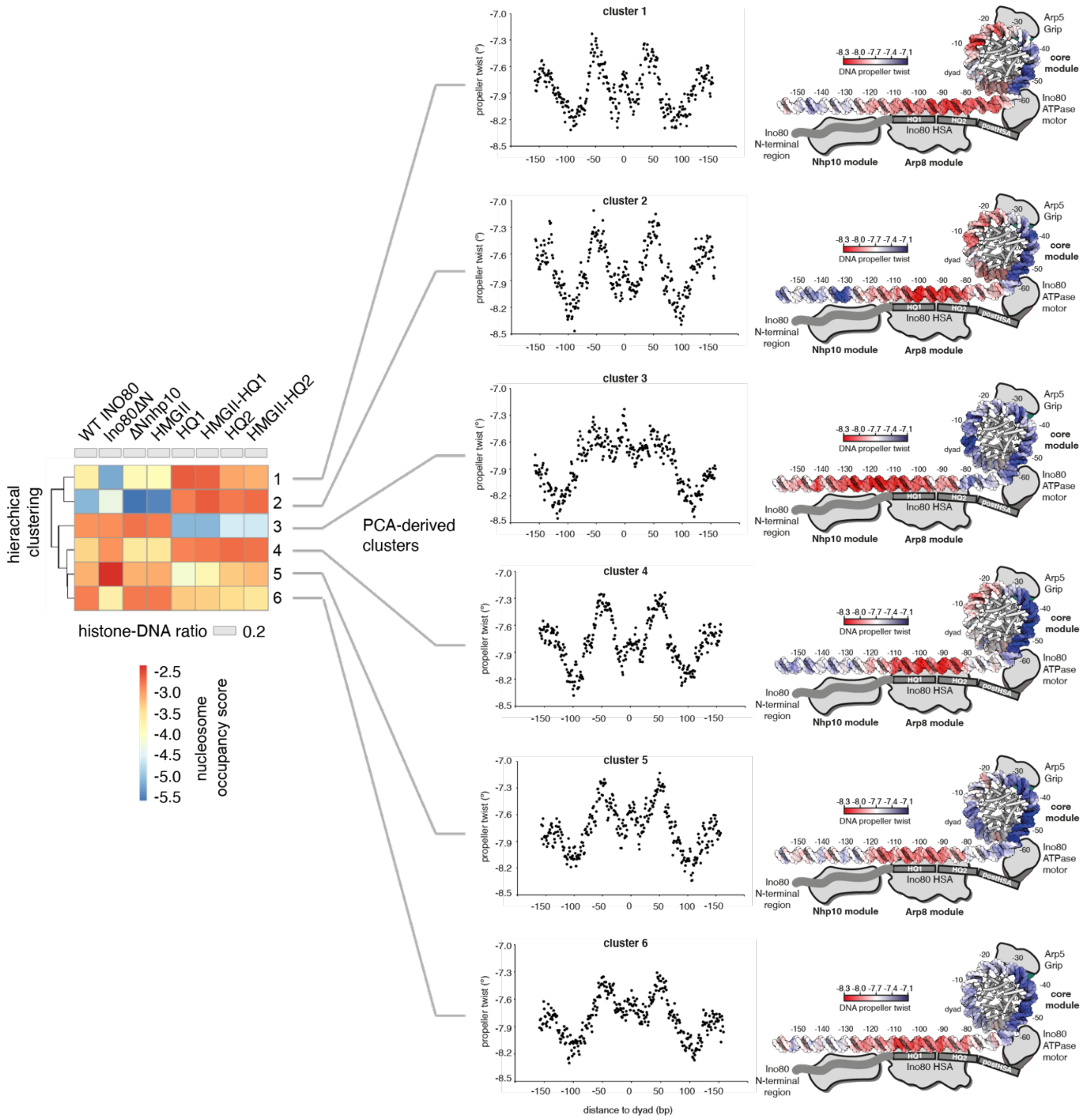

Supplementary Figure 3. PCA/clustering and DNA shape analysis for WT and mutant INO80. The same heat map as in Figure 5c is shown, but now with DNA propeller twist shape profiles and mapping to a model of linker and nucleosome core DNA as in Figure 4a,b for nucleosomal DNA sequences in each cluster.

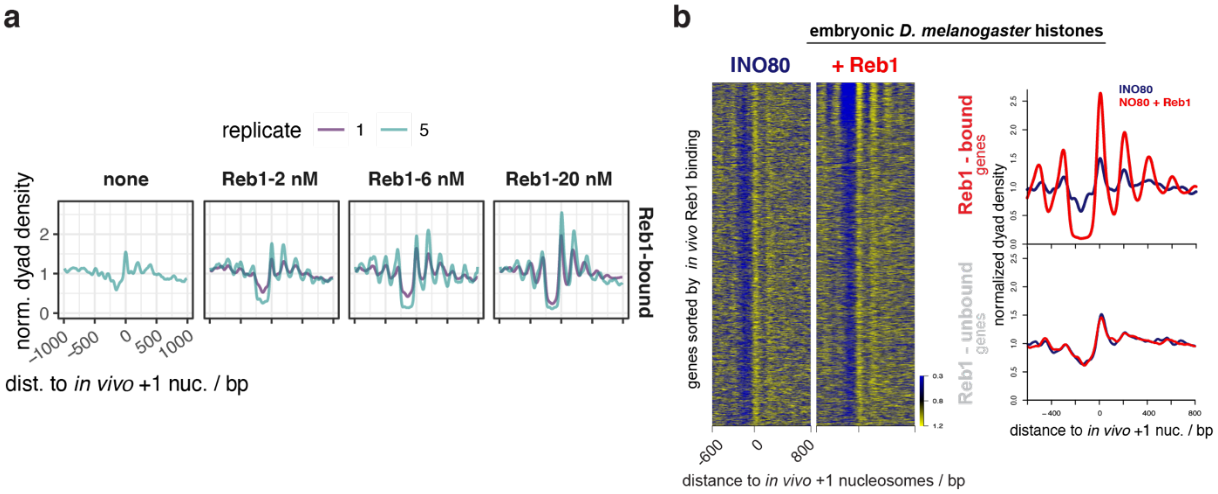

Supplementary Figure 4. Nucleosome positioning by INO80 in presence of Reb1. a Composite plots of MNase-seq data as in Figure 6a,b, but for individual replicates and only for genes with promoter Reb1-sites (Reb1-bound, same as red shading in Figure 6a) and also including SGD chromatin incubated with INO80 in the absence of Reb1 (none). **b** As Figure 6a,b, but single replicate for SGD chromatin with embryonic *D. melanogaster* histones at histone-to-DNA mass ratio of 0.4 remodeled by recombinant WT *S. cerevisiae* INO80 without (INO80) or with 20 nM Reb1 (+ Reb1).

Supplementary Table 1. **Plasmids and primers used.**

REAGENT or RESOURCE	SOURCE	IDENTIFIER
Recombinant DNA		
pET21_601	Daniela Rhodes, MRC LMB, UK	N/A
pFBDM	pFastBacDUAL Invitrogen, Trowitzsch, Bieniossek et al. 2010)	Addgene 110738
pFBDM-Rvb1-Rvb2-Ies6-Arp5-Ino80_HQ1	Knoll et al., 2018 (reference 3)	N/A
pFBDM-Rvb1-Rvb2-Ies6-Arp5-Ino80_HQ2	Knoll et al., 2018 (reference 3)	N/A
pFBDM-Ies1-Ies3-Ies5-ΔNhp10	This work	N/A
pFBDM-Ies1-Ies3-Ies5-Nhp10(HMGII)	This work	N/A
pFBDM-Rvb1-Rvb2-Ies6-Arp5-Ino80ΔN	This work	N/A
pET21b	Novagen	Addgene 72327
pET21b-Reb1	This work	N/A
Sequence based Reagents		
Primer Ino80-HQ1 for	CAACACCTATACAACTATTTGGAA AGACATGGCTCAGC	Metabion
Primer Ino80-H1Q rev	CATAGCCTCCTTTTCAATCTTCTT CTTTAGATCTCTTTC	Metabion
Primer Ino80_H2Q for	CAACACCTATACAACTATTTGGAA AGACATGGCTCG	Metabion
Primer ino80_H2Q rev	CATAGCCTCCTTTTCAATTTGCTG CTGTAGATCCTG	Metabion
Primer Nhp10-HMG for	GGCCTCCTTCAAACAGGAACTATT GACGAAGCCATTTC	Metabion
Primer Nhp10-HMG rev	CAGATCTCTTGTCACGTCCAGAGA GCCATTCTG	Metabion
Primer Ino80$^{\Delta N}$ for	GCCTACGTCGACATGGCCCGTGC TATCCAGAGGCATT	Metabion
Primer Ino80$^{\Delta N}$ rev	CTGGATAGCACGGGCCATGTCGA CGTAGGCCTTTGAATTCCG	Metabion

Supplementary References

1. Ayala, R. et al. Structure and regulation of the human INO80-nucleosome complex. *Nature* **556**, 391-395 (2018).
2. Eustermann, S. et al. Structural basis for ATP-dependent chromatin remodelling by the INO80 complex. *Nature* **556**, 386-390 (2018).
3. Knoll, K.R. et al. The nuclear actin-containing Arp8 module is a linker DNA sensor driving INO80 chromatin remodeling. *Nat Struct Mol Biol* **25**, 823-832 (2018).
4. Oberbeckmann, E. et al. Ruler elements in chromatin remodelers set nucleosome array spacing and phasing. *Nature Communications* (2021).
