# Supplementary material for: Genome information processing by the INO80 chromatin remodeler positions nucleosomes
Source: Nat Commun. 2021 May 28;12:3231. doi: 10.1038/s41467-021-23016-z (PMC8163841; doi:10.1038/s41467-021-23016-z)
Supplement: Supplementary file 2 — Description of Additional Supplementary Files [file 41467_2021_23016_MOESM2_ESM.pdf]

Description of Additional Supplementary Files

File Name: Supplementary Data 1
Description: Experimental information summary for each reconstituted chromatin sample used in this study for composite plots and heatmaps. Each replicate represents an individually assembled chromatin on a different day. The processed data file can be downloaded at GEO (GSE145093).

File Name: Supplementary Data 2
Description: Experimental information summary for each reconstituted chromatin sample used for PCA/clustering and DNA shape/mechanics analysis. Each replicate represents an individually assembled chromatin on a different day. The processed data file can be downloaded at GEO (GSE145093).
